# Educational Attainment Decreases the Risk of COVID-19 Severity in the European Population: A Two-Sample Mendelian Randomization Study

**DOI:** 10.3389/fpubh.2021.673451

**Published:** 2021-06-03

**Authors:** Masahiro Yoshikawa, Kensuke Asaba

**Affiliations:** ^1^Division of Laboratory Medicine, Department of Pathology and Microbiology, Nihon University School of Medicine, Tokyo, Japan; ^2^Department of Computational Diagnostic Radiology and Preventive Medicine, The University of Tokyo Hospital, Tokyo, Japan

**Keywords:** Mendelian randomization, COVID-19, SARS-CoV-2, educational attainment, years of schooling

## Abstract

Observational studies have reported that the severity of COVID-19 depends not only on physical conditions but also on socioeconomic status, including educational level. Because educational attainment (EA), which measures the number of years of schooling, is moderately heritable, we investigated the causal association of EA on the risk of COVID-19 severity using the Mendelian randomization (MR) approach. A two-sample MR analysis was performed using publicly available summary-level data sets of genome-wide association studies (GWASs). A total of 235 single-nucleotide polymorphisms (SNPs) were extracted as instrumental variables for the exposure of EA from the Social Science Genetic Association Consortium GWAS summary data of 766,345 participants of European ancestry. The effect of each SNP on the outcome of COVID-19 severity risk was obtained from the GWAS summary data of 1,059,456 participants of European ancestry gathered from the COVID-19 Host Genetics Initiative. Using inverse variance weighted method, our MR study shows that EA was significantly associated with a lower risk of COVID-19 severity (odds ratio per one standard deviation increase in years of schooling, 0.540; 95% confidence interval, 0.376–0.777, *P* = 0.0009). A series of sensitivity analyses showed little evidence of bias. In conclusion, we show for the first time using a two-sample MR approach the associations between higher EA and the lower risk of COVID-19 severity in the European population. However, the genetic or epidemiological mechanisms underlying the association between EA and the risk of COVID-19 severity remain unknown, and further studies are warranted to validate the MR findings and investigate underlying mechanisms.

## Introduction

The coronavirus disease 2019 (COVID-19), caused by a novel coronavirus SARS-CoV-2 (severe acute respiratory syndrome coronavirus 2), was originally reported as an outbreak of atypical pneumonia cases in Wuhan in the Hubei Province of China in December 2019. As of March 2021, the COVID-19 death toll has topped 2.8 million worldwide according to the World Health Organization (1). Serious COVID-19 patients have pneumonia with hypoxia and may be critical with acute respiratory distress syndrome, pulmonary fibrosis, and other organ failures (2).

Observational studies report that the severity of COVID-19 depends not only on physical conditions such as age, cardiovascular disease, and obesity (3–8) but also on socioeconomic status (SES) indicators such as lower incomes and lower educational level among various populations (9–12). In the European population, lower education level was associated with a higher risk of severe COVID-19 cases that were confirmed either at emergency departments or as inpatients and, therefore, likely reflect severe illness as well as a higher risk of asymptomatic COVID-19 cases in a prospective cohort study using UK Biobank data (9). However, traditional observational studies lacking randomization designs are generally prone to bias by various factors, including confounders and reverse causations (13).

Mendelian randomization (MR) is an epidemiological method that mimics the design of randomized controlled studies using single-nucleotide polymorphisms (SNPs) as instrumental variables (IVs) and examines the causal effects of a risk factor on an outcome of interest. Because genetic variants, such as SNPs, are randomly assigned at conception according to Mendel's law, MR studies are not influenced by confounders or reverse causations and can overcome limitations of observational studies (13). Educational attainment (EA) is highly affected by environmental and social factors but is also moderately heritable as shown by genome-wide association studies (GWASs) (14, 15). Therefore, we were motivated to investigate in this study whether EA had a causal effect on the risk of COVID-19 severity using the MR approach.

## Methods and Materials

### Study Design and Data Sources

We conducted a two-sample MR study using publicly available summary statistics from two GWASs to investigate whether EA was associated with risk of COVID-19 severity. In MR analysis, SNPs from the exposure data set are used as IVs. IVs must satisfy the following three assumptions: The IVs are associated with the exposure (IV assumption 1), the IVs affect the outcome only *via* the exposure (IV assumption 2), and the IVs are not associated with measured or unmeasured confounders (IV assumption 3) (16). For the exposure data set of EA, which measured the number of years of schooling that individuals had completed, the SNPs were obtained from the Social Science Genetic Association Consortium's GWAS summary data of 766,345 participants of European ancestry (13), which was a meta-analysis of 70 discovery cohorts (excluding 23andMe) as shown in Supplementary Table 1. This data set was publicly available from the MRC IEU Open GWAS database (17) and MR-Base (18) given as GWAS-ID of “ieu-a-1239.” For the outcome data set of the risk of COVID-19 severity, the SNPs were obtained from summary-level GWAS data of COVID-19-hg GWAS meta-analyses (round 5) including 14 studies, but excluding the UK Biobank, with a total of 1,059,456 participants (4,792 very severe respiratory confirmed COVID-19 cases and 1,054,664 controls) of European ancestry by the COVID-19 Host Genetics Initiative (19) (Supplementary Table 1), which was released on January 18, 2021, and was also publicly available (20). Very severe respiratory confirmed COVID-19 cases were defined as hospitalization for laboratory confirmed SARS-CoV-2 infection with death or respiratory support (20).

### Selection of Instrumental Variables

The SNPs were selected from the exposure GWAS summary data as IVs by clumping together all SNPs that were associated with EA at a genome-wide significance threshold (*P* < 5.0 × 10^−8^) and were not in linkage disequilibrium (*r*^2^ < 0.01 and distance > 10,000 kb) with the other SNPs. Palindromic SNPs with minor allele frequency > 0.42 were excluded from the analyses (16, 21). As a sensitivity analysis, we also excluded all palindromic SNPs regardless of minor allele frequencies (22). We studied only SNPs that were present in both the exposure and outcome GWAS data sets and did not include proxy SNPs in the analysis (22, 23). To evaluate the strength of the IVs, we calculated the *F*-statistic of each SNP using the following formula: *F*-statistic = *R*^2^ × (*N* − 2)/(1 − *R*^2^), where *R*^2^ is the variance of the phenotype explained by each genetic variant in exposure, and *N* is the sample size. *R*^2^ was calculated using the following formula: *R*^2^ = 2 × (Beta)^2^ × EAF × (1−EAF)/[2 × (Beta)^2^ × EAF × (1−EAF) + 2 × (SE)^2^ × *N* × EAF × (1−EAF)], where Beta is the per allele effect size of the association between each SNP and phenotype, EAF is the effect allele frequency, and SE is the standard error of Beta (24). IVs with an *F*-statistic <10 were regarded as weak instruments (25).

### Two-Sample Mendelian Randomization

The Wald ratio, which estimates causal effect for each IV, was calculated as the ratio of Beta for the corresponding SNP in the outcome data set divided by Beta for the same SNP in the exposure data set (26). Our main approach was to conduct a meta-analysis of each Wald ratio by inverse variance weighted (IVW) method using multiplicative random-effects model to estimate overall causal effect of the exposure on the outcome. The causal effects were calculated as the odds ratio (OR) for the risk of COVID-19 severity per one standard deviation (SD) increase in years of schooling (one SD is equivalent to 4.2 years) (15, 27). In addition, we conducted sensitivity analyses by MR-Egger regression, weighted median method, MR-PRESSO (Mendelian Randomization Pleiotropy RESidual Sum and Outlier) global test, and leave-one-out sensitivity analysis. The MR-Egger regression method is used to assess horizontal pleiotropy of IVs. When IV assumption 2 is violated, horizontal pleiotropy occurs, and MR-Egger regression intercept significantly differs from zero (28, 29). The weighted median method provides a valid causal estimate when more than half of the instrumental SNPs satisfy the IV assumptions (24). The MR-PRESSO global test investigates whether there are outlier SNPs whose variant-specific causal estimates differ substantially from those of other SNPs (30, 31). Leave-one-out sensitivity analysis was conducted to assess the reliability of the IVW method by removing each SNP from the analysis and reestimating the causal effect (31). Moreover, among SNPs associated with EA, we searched for SNPs associated with *P* < 5.0 × 10^−8^ with pleiotropic effects on body mass index (BMI), smoking, and other SES using the web tool PhenoScanner (version 2) (32, 33). The heterogeneity was also measured between the causal estimates across all SNPs in the IVW method calculating Cochran's *Q* statistic and *I*^2^ statistic (34). Low heterogeneity provides more reliability for a causal effect (35). We conducted all the two-sample MR analyses using “TwoSampleMR” package (version 0.5.5) in *R* (version 4.0.3) (36). A *P*-value below 0.05 was considered statistically significant in all statistical analyses.

## Results

In total, 235 instrumental SNPs were identified for both EA and the risk of COVID-19 severity GWAS data sets. The characteristics of all the SNPs included in our analysis are shown in Supplementary Table 2. The *F*-statistic of every instrument was >29, thus suggesting that weak instrument bias was unlikely.

**Figure 1:**
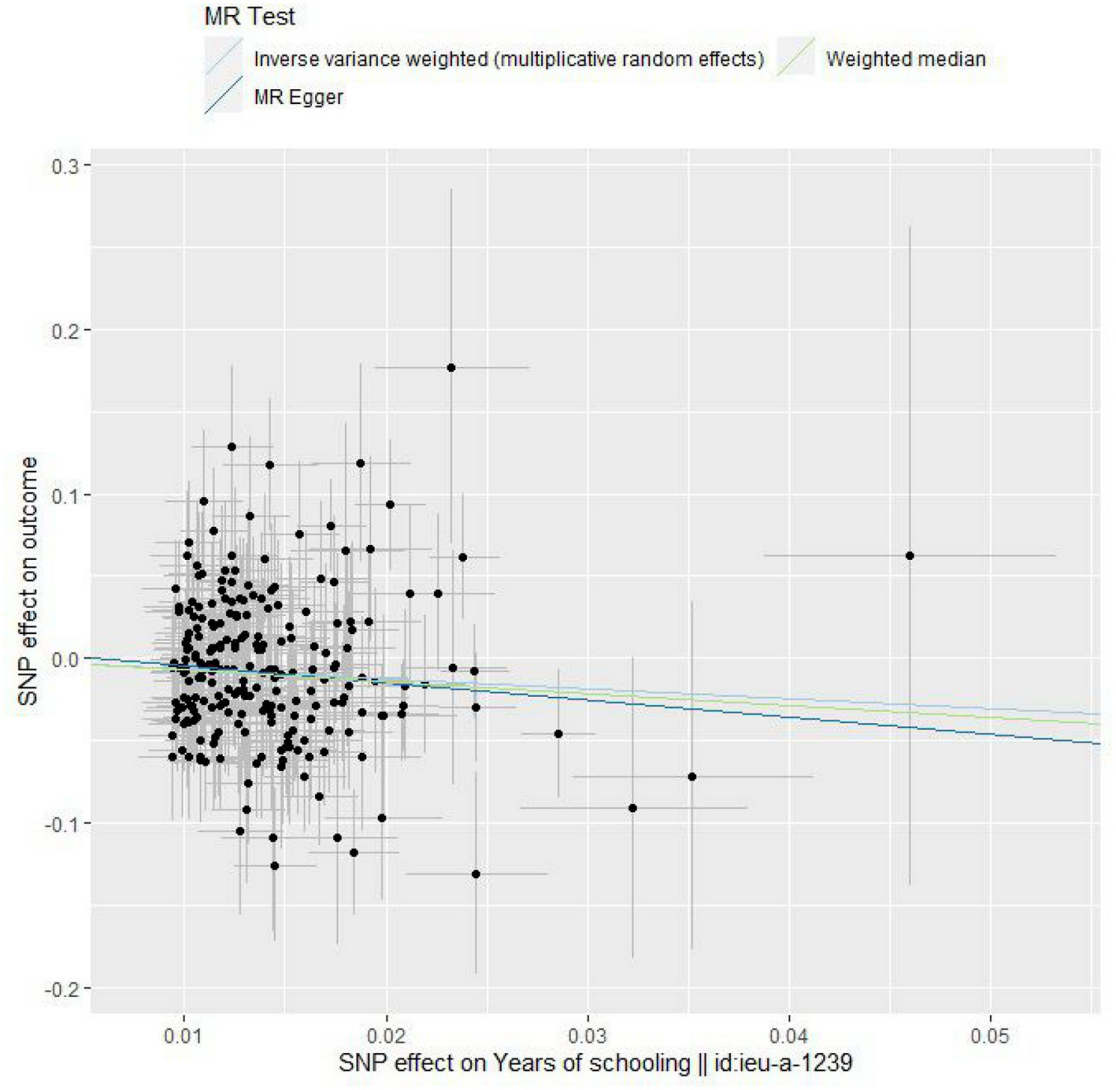
Scatter plots. Each black point representing an SNP is plotted in relation to the effect size of the SNP on years of schooling (x-axis) and on the risk of COVID-19 severity (y-axis) with corresponding standard error bars. The slope of each line corresponds to the causal estimate using inverse variance weighted (light blue), weighted median (green), and MR-Egger regression (blue) method.

The IVW method showed that EA was significantly associated with a lower risk of COVID-19 severity [OR per 1-SD increase in years of schooling, 0.540; 95% confidence interval (CI), 0.376–0.777; *P* = 0.0009] in the European population (Table 1, Figure 1, and Supplementary Figure 1). Cochran's *Q* statistic and *I*^2^ statistic for the IVW method were 261.9 (*P* = 0.102) and 0.110, indicating low heterogeneity and more reliability for the causal effect. Other MR methods also showed overall consistent protective effects for EA on the risk of COVID-19 severity although the MR-Egger regression estimate did not have statistical significance (Table 1 and Figure 1). However, when *I*^2^ statistics are much <1, no measurement error assumption is violated and MR-Egger regression tends to underestimate the causal effect (34). In fact, Cochran's *Q* statistic and *I*^2^ statistic for our MR-Egger regression were 261.5 (*P* = 0.097) and 0.113 (much <1), respectively. The MR-Egger intercept was 0.006 (*P* = 0.548), indicating little evidence of horizontal pleiotropy. The weighted median method indicated that more than half of the instrumental SNPs in our analysis satisfied the IV assumptions. The funnel plot showed general symmetry, suggesting little evidence of heterogeneity or horizontal pleiotropy (Supplementary Figure 2). MR-PRESSO global test (*P* = 0.115) and leave-one-out sensitivity analysis suggested the lack of an outlier SNP whose variant-specific causal estimate differed substantially from those of other SNPs (Table 1 and Supplementary Figure 3). We excluded all palindromic SNPs regardless of minor allele frequencies from the IVW method, and then we obtained a comparable result to that of the original IVW method (OR, 0.540; 95% CI, 0.366–0.796, *P* = 0.0019; number of instrumental SNPs; 209). Our search using PhenoScanner identified 17 SNPs that were associated with BMI (rs10073890, rs11123818, rs13090388, rs1334297, rs1566085, rs1618725, rs1689510, rs1964927, rs2725370, rs2820314, rs4787457, rs56391344, rs62444881, rs66568921, rs67890737, rs9372625, rs9384679), three SNPs that were associated with smoking traits (rs10240905 with pack years adult smoking as proportion of life span exposed to smoking, rs2179152 with pack years of smoking, and rs66568921 with ever smoked), and six SNPs that were associated with other SES traits (rs1008078 with Townsend deprivation index at recruitment, rs13090388, rs34316, and rs9372625 with job involving heavy manual or physical work, rs1391438 and rs2971970 with job involving mainly walking or standing), respectively. We excluded the 17 SNPs that were associated with BMI from the IVW method, and then we obtained a comparable result to that of the original IVW method (OR, 0.550; 95% CI, 0.373–0.810, *P* = 0.0025; number of instrumental SNPs; 218) (see section Discussion). When we excluded the three SNPs that were associated with smoking traits from the IVW method, we obtained a comparable result to that of the original IVW method (OR, 0.557; 95% CI, 0.386–0.803, *P* = 0.0018; number of instrumental SNPs; 232). Similarly, we excluded the six SNPs that were associated with other SES traits from the IVW method, and then we obtained a comparable result to that of the original IVW method (OR, 0.536; 95% CI, 0.372–0.773, *P* = 0.0009; number of instrumental SNPs; 229). Moreover, when we excluded all 23 SNPs that were associated with BMI, smoking traits, and other SES traits (three SNPs overlapped), we obtained a comparable result to that of the original IVW method (OR, 0.565; 95% CI, 0.382–0.835, *P* = 0.0042; number of instrumental SNPs; 212).

**Table 1:** MR results of the causal effect of EA on the risk of COVID-19 severity.

**IVW method**	**Weighted median method**	**MR-Egger regression method**		**Heterogeneity (IVW)**	**MR-PRESSO global test**
**OR (95% CI)**	**OR (95% CI)**	**OR (95% CI)**	**Intercept**	**Cochran's *Q***	
***P*-value**	***P*-value**	***P*-value**	***P*-value**	***P*-value**	***P*-value**
0.540 (0.376–0.777)	0.484 (0.283–0.826)	0.353 (0.084–1.483)	0.006	261.9	
*P* = 0.0009	*P* = 0.008	*P* = 0.156	*P* = 0.548	*P* = 0.102	*P* = 0.115

*MR, Mendelian randomization; COVID-19, coronavirus disease 2019; EA, educational attainment; IVW, inverse variance weighted; OR, odds ratio; CI, confidence interval*.

We noticed some possible overlap between the exposure GWAS participants and the outcome GWAS participants as shown in Supplementary Table 1. This might have led to bias in the causal estimate of EA on the risk of COVID-19 severity, but the bias was unlikely to be substantial because the possible overlap was small as discussed below.

## Discussion

To our knowledge, this is the first MR study to investigate the association between EA and the risk of COVID-19 severity. Observational studies report that a lower level of education influences the severity of COVID-19 among various populations (9–12). In the European population, those who had no qualification (equivalent to seven years of education) (37) had a higher risk of severe COVID-19 (i.e., a positive test for SARS-CoV-2 in a hospital setting either at emergency departments or as inpatients) than those who had college or university degree (equivalent to 20 years of education) (37) in fully adjusted model [risk ratio (RR), 1.58; 95% CI, 1.25–1.99; *p* < 0.001] in a prospective cohort study using UK Biobank data (9). Our two-sample MR approach supported, with little evidence of bias, the causal effect of higher EA on the risk of COVID-19 severity (OR, 0.540; 95% CI, 0.376–0.777; *P* = 0.0009) in the European population, which was consistent with the cohort study. In other populations, a risk-adjusted model of a large cohort, including 62,298 COVID-19 deaths, showed that lower education levels were strongly associated with the level of COVID-19 fatalities per 100,000 persons (rate ratio, 1.08; 95% CI, 1.05–1.11; *P* < 0.0001) in severely distressed counties in the United States (10). Another study in the United States showed that education level with a bachelor's degree was associated with a lower rate of mortality due to COVID-19 (estimate, −0.246; 95% CI, −0.388 to −0.103; *P* = 0.0008) across various ethnicities in the seven most affected states (11). In São Paulo, Brazil, among patients under 60 years of age and living in areas with the lowest percentage (below 8.61%) of the population with a university degree, COVID-19 mortality was four times higher than that among those living in areas with the highest percentage (over 34.80%) of population with a university degree (rate ratio, 4.02; 95% CI, 3.42–4.72) (12). However, our MR analysis was based on populations of European ancestry, and the findings are unlikely to be generalized to other populations and ethnicities.

In our MR analysis, underlying genetic or epidemiological mechanisms of how EA lowered the risk of COVID-19 severity remain unknown. Therefore, although a range of sensitivity analyses indicated the robustness of our MR findings, we must pay careful attention to the possibility of unmeasured horizontal pleiotropy of genetic IVs for EA. Observational studies showed that higher EA was associated with decreased prevalence of smoking, physical inactivity, obesity, hypertension, and hypercholesterolemia (38). We infer that other risk factors, including BMI and lifetime smoking, were related to the causal effect of EA on the risk of COVID-19 severity in our analysis for the following reasons. First, MR studies have shown that EA has causal effects on decrease of BMI (39, 40). Second, MR studies have shown that BMI has a causal effect on the risk of COVID-19 severity (29, 41, 42). Consistent with the MR results showing the effect of BMI on the risk of COVID-19 severity, the risk-adjusted model showed that, in addition to the two socioeconomic factors of low level of education and a proportionally larger Black population, obesity was the only physical risk factor in the U.S. cohort (10). Other observational studies also have reported that BMI is a risk factor for hospital admission, disease severity, and in-hospital mortality due to COVID-19 (5–8). Consistently, our search using PhenoScanner identified 17 SNPs that were associated with both EA and BMI with *P* < 5.0 × 10^−8^. Similarly, MR studies have shown that EA has causal effects on increased lifetime smoking (39) and that lifetime smoking has a causal effect on the risk of COVID-19 severity (41) although the associations between smoking and the risk of COVID-19 remain controversial in observational studies (41). Therefore, the causal effect of EA on the risk of COVID-19 severity may be at least partly mediated through increases of BMI and lifetime smoking. Even if that is the case, EA would remain an intervention target for COVID-19 severity (43). In fact, when we excluded the 17 SNPs that were associated with BMI and the three SNPs that were associated with smoking traits, we obtained comparable results to the result of the original IVW method as described above. This supports the idea that EA remains an intervention target for COVID-19 severity because EA lowered the risk of COVID-19 severity to some extent independently of the effects of BMI and smoking.

Epidemiologically, the protective effect of EA on the risk of COVID-19 severity may be related to the social benefit of education. Observational studies showed that lower EA as well as other SES was associated with disparities in medical care (44). For example, counties in the United States with a higher percentage of people below the poverty level had a significantly lower percentage of the population with higher education as well as a lower percentage of people insured (11), and counties in the United States with higher income and education, a lower rate of disability, and a higher rate of the insured population were at a lower risk of COVID-19 mortality (11). However, we must pay attention to interpret the causal association between lower EA and the risk of COVID-19 severity because it remains unclear epidemiologically whether less educated people are more likely to develop severe COVID-19 symptoms. In other words, there is possibility that less educated people are more likely to be socioeconomically disadvantaged and to have an increased risk of SARS-CoV-2 transmission due to poor housing, overcrowding, and low-paid essential jobs that make social distancing more challenging (45). As a result of higher COVID-19 incidence, they may have a higher risk of COVID-19 severity. Ascertainment bias could also arise due to differential healthcare seeking, differential testing, and differential prognosis (9). We could not conduct an MR analysis investigating a causal effect of EA on the risk of COVID-19 incidence as described below. However, the prospective cohort study using UK Biobank data showed that both lower education and area-level socioeconomic deprivation by the Townsend index were associated with having a positive test including asymptomatic COVID-19 [RR 1.46 for no qualifications vs. degree (95% CI 1.19–1.79), and RR 1.39 for most deprived quartile vs. least (95% CI 1.12–1.71)] as well as a higher risk of testing positive in hospital (i.e., severe COVID-19 cases) [RR 1.58 for no qualifications vs. degree (95% CI 1.25–1.99), and RR 1.54 for most deprived quartile vs. least (95% CI 1.21–1.97)] in the fully adjusted model (9). The authors discussed that there remained the possibility that some socioeconomic groups had a poorer prognosis and were, therefore, more likely to be admitted to hospital and, therefore, to be tested (9).

The present study includes the following strengths. First, the samples used were gathered across populations with the same European ancestries, reducing substantial bias in our study. Among different genetic ancestries, effect sizes and allele frequencies can differ and lead to substantial bias (24). Second, we used the publicly available GWAS data sets with the largest sample sizes hitherto for both the exposure and outcome data sets. *F*-statistics were also large enough for weak instrument bias to be unlikely. Third, a range of sensitivity analyses relaxed the IV assumptions and supported the robustness of our MR findings.

However, we must pay attention to several major limitations. First, in the Geisinger Health System study, the participants in the exposure GWAS may have overlapped with the participants in the outcome GWAS as shown in Supplementary Table 1. This might have led to bias in the causal estimate of EA on the risk of COVID-19 severity (46). It was difficult for us to exclude the Geisinger Health System study because we used summary-level data for the exposure and outcome data sets. However, the participants in the Geisinger Health System study represented only 1.9% (14,562 out of 766,344) of those in the exposure GWAS data set. Moreover, the participants in the Geisinger Health System_EUR study represented only 1.2% (53 out of 4,392) of the severe COVID-19 cases, and most of them (10.7%, 112,862 out of 1,054,664) were controls in the outcome GWAS data set. If the data sets are of different sizes, the percentage overlap should be taken with respect to the larger data set (46). Therefore, vast majority of the participant overlap in the outcome GWAS data set occurred, if at all, among the controls. In that situation, the bias is unlikely to be substantial, and unbiased causal estimates are expectedly obtained in two-sample MR studies (41, 46). On the other hand, we could not conduct an MR analysis investigating a causal effect of EA on the risk of COVID-19 incidence because the summary-level GWAS data of COVID-19 incidence (i.e., 32,494 SARS-CoV-2 infection cases and 1,316,207 controls in the European population) by the COVID-19 Host Genetics Initiative (19, 20) had possible participant overlap [at most, 16.2% (5,270 out of 32,494) of the SARS-CoV-2 infection cases in the deCODE_EUR, the Geisinger Health System_EUR, and the Netherlands Twin Register_EUR studies] with the EA GWAS data set that could cause substantial bias (46) (Supplementary Table 1). Second, our MR findings might be affected by unmeasured horizontal pleiotropy as described above. As is the often the case with many MR studies, strictly satisfying all the IV assumptions can be challenging (47). Third, our MR analysis was based on populations of European ancestry, and the findings are unlikely to be generalized to other populations and ethnicities. Fourth, we could not conclude that the risk of COVID-19 severity could decrease simply by increasing years of schooling because the underlying genetic or epidemiological mechanisms remain unknown.

In conclusion, we have shown for the first time using a two-sample MR approach the associations between higher EA and the lower risk of COVID-19 severity in the European population that observational studies have reported. However, genetic or epidemiological mechanisms underlying the association between EA and the risk of COVID-19 severity remain unknown, and further studies are warranted to validate our MR findings and investigate underlying mechanisms.

## Data Availability Statement

Publicly available datasets were analyzed in this study. This data can be found here: https://gwas.mrcieu.ac.uk/datasets/ieu-a-1239/, https://www.covid19hg.org/results/r5/.

## Ethics Statement

Ethical approval was not provided for this study on human participants because we used only publicly available GWAS summary datasets. Written informed consent was not provided because we used only publicly available GWAS summary datasets.

## Author Contributions

MY designed this study, analyzed data, and wrote the draft of the manuscript. MY and KA discussed and reviewed the manuscript critically. Both authors contributed to the article and approved the submitted version.

## Conflict of Interest

The authors declare that the research was conducted in the absence of any commercial or financial relationships that could be construed as a potential conflict of interest.

## Supplementary Material

Supplementary Figure 1 Forrest plots. Each black point represents the causal estimate of each SNP on the risk of COVID-19 severity per increase in years of schooling, and red points show the combined causal estimates using IVW and MR-Egger regression methods with horizontal lines denoting 95% confidence intervals.

Supplementary Figure 2 Funnel plots. Each black point representing an SNP is plotted in relation to the estimate of years of schooling on the risk of COVID-19 severity (x-axis) and the inverse of standard error (y-axis). Vertical lines show the combined causal estimates using IVW (light blue) and MR-Egger regression (blue) methods.

Supplementary Figure 3 Leave-one-out sensitivity analysis. Each black point represents the combined causal estimates on the risk of COVID-19 severity per increase in years of schooling using IVW methods with horizontal lines denoting 95% confidence intervals after removing the corresponding SNP from the analysis.

Supplementary Table 1 Contributing studies of the exposure GWAS data and the outcome GWAS data.

Supplementary Table 2 The characteristics of all the SNPs included in our analysis.
